# Endoscopic and video capsule endoscopic observation of *Yersinia* enterocolitis

**DOI:** 10.1002/deo2.242

**Published:** 2023-04-27

**Authors:** Kyouko Marubashi, Hitoshi Takagi, Tadatake Wakagi, Satoshi Takakusagi, Youzou Yokoyama, Kazuko Kizawa, Takashi Kosone, Toshio Uraoka

**Affiliations:** ^1^ Department of Gastroenterology and Hepatology Kusunoki Hospital Gunma Japan; ^2^ Department of Gastroenterology and Hepatology Gunma University Graduate School of Medicine Gunma Japan

**Keywords:** biopsy culture, Crohn's disease, granulomatous inflammation, video capsule endoscopy, *Yersinia* enterocolitis

## Abstract

A woman in her late 20s suffered from epigastralgia following lower abdominal pain with diarrhea. Kampo medicine relieved the complaints, but the pain recurred a month later. She had immigrated from Vietnam to Japan 6 months before the onset of the abdominal pain. Blood test findings were almost within normal limits, except for mild C‐reactive protein elevation and positive *Helicobacter pylori* antibody findings. Colonoscopy revealed an edematous cobblestone‐like appearance at the end of the ileum with irregular ulceration mimicking Crohn's disease. Video capsule endoscopy was performed to detect lesions in the small intestine and demonstrated irregular ileal ulcer, reminiscent of Crohn's disease. A biopsy performed during colonoscopy demonstrated granulomatous inflammation with a moderate accumulation of plasma cells and mononuclear cells. The bacterial culture of the biopsy specimen proved the growth of *Yersinia enterocolitica*. Levofloxacin 500 mg for 7 days rapidly relieved abdominal pain. *Yersinia* enterocolitis is rare in developed countries, but as a differential diagnosis for Crohn's disease, it is important to treat. This is the first case report of the video capsule endoscopy findings of *Yersinia* enterocolitis. Video capsule endoscopy can help to confirm the spread of the lesions of *Yersinia* enterocolitis.

## INTRODUCTION

*Yersinia* enteritis is typically caused by the Gram‐negative anaerobic rods *Yersinia enterocolitica* (YE) and *Yersinia pseudotuberculosis*.^1^ *Yersinia* is transmitted to humans via water, food, soil, and animals.^2^ The most important reservoirs are rodents, domestic animals, and birds. Pork products as well as minced meat can be contaminated by insects spreading the contamination to other meat cuts during slaughter.^2^

Although yersiniosis seemed to be rare in developed countries, a significant number of YE infections have been reported in those areas of temperate zones.^3^ *Yersinia* enterocolitis is sometimes difficult to differentiate from Crohn's disease (CD), as both involve gastrointestinal symptoms and endoscopic findings such as irregular ulceration around the terminal ileum.^1^, ^2^

In connection with food poisoning, *Yersinia* is an important pathogen both globally^3^ and in Japan.^4^ Advances in video capsule endoscopy (VCE) have resulted in the detailed depiction of ileal lesions of various etiologies.^5^

The present case involved *Yersinia* enterocolitis which was eventually identified by bacterial culture using a biopsy specimen. We herein report for the first time the typical endoscopic and VCE findings for *Yersinia*‐induced terminal ileitis and colitis.

## CASE REPORT

**FIGURE 1:**
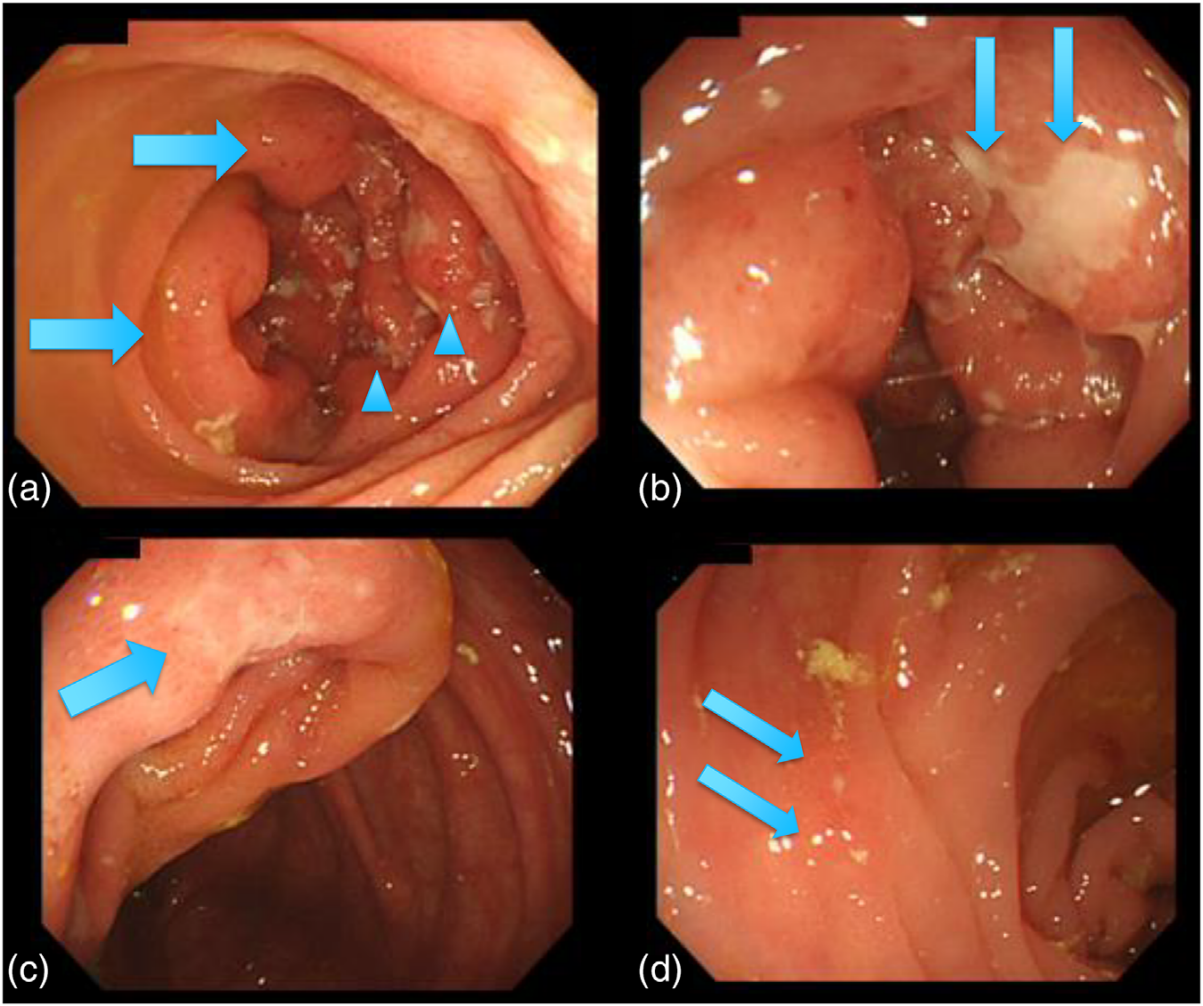
Colonoscopic findings the day after the first admission. (a) An edematous cobblestone appearance at the end of the ileum (thick arrows) with longitudinal irregular ulceration mimicking Crohn's disease (arrowheads). (b) Close‐range view of the ulcers (thin arrows). (c) Mucosal erosions with a white coat on Bauhin's valve. (d) Smaller points of erosive redness were also observed in the ascending colon.

**FIGURE 2:**
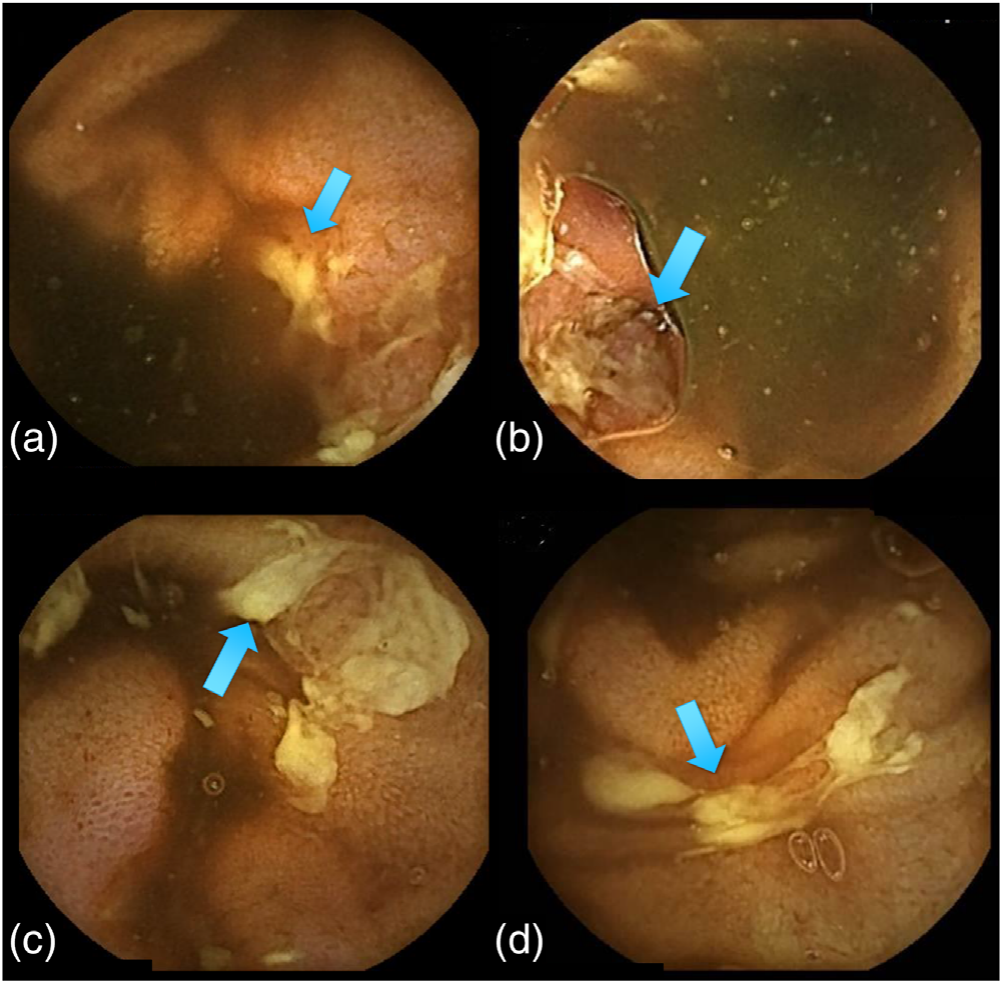
At 4 days after colonoscopy, video capsule endoscopy (VCE) revealed more lesions at the oral side of the ileum end, reminiscent of Crohn's disease (CD) (a–d, arrow).

**FIGURE 3:**
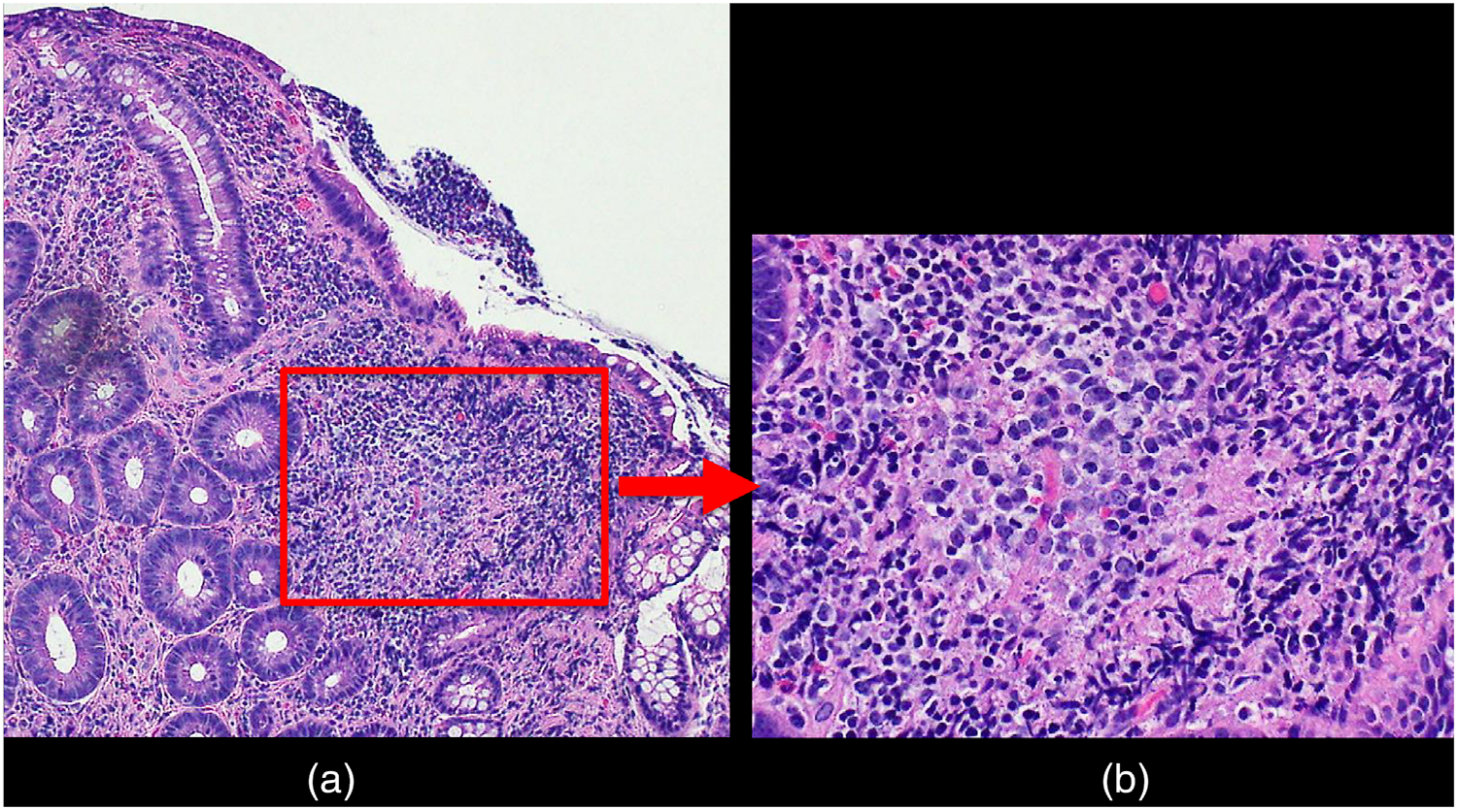
A biopsy performed during colonoscopy demonstrated granulomatous inflammation with a moderate accumulation of plasma cells and mononuclear cells. (a) 100x, Hematoxylin‐Eosin (HE) staining, (b) 400x, HE staining of the selected part of (a).

A woman in her late 20s visited our hospital because of epigastralgia following lower abdominal pain with diarrhea. Kampo medicine relieved the complaints, but the pain recurred a month later. She had immigrated to Japan from Vietnam 6 months before the onset of the abdominal pain. No obvious abnormalities in her physical findings were observed, except for paraumbilical tenderness. Blood test findings were almost within normal ranges regarding the blood cell count and chemistry and serological markers for infection, aside from mild C‐reactive protein elevation (0.7 mg/dl) and positive findings for *Helicobacter* antibody. Enhanced computed tomography did not demonstrate specific findings in the abdomen including the ileocecal region on the day of the first visit. Upper gastrointestinal endoscopy revealed atrophic gastritis reminiscent of helicobacter pylori infection but no other specific lesions like CD were observed. Colonoscopy performed the day after her first visit revealed an edematous cobblestone appearance at the end of the ileum with irregular ulceration without longitudinal arrangement mimicking CD (Figure 1a,b). Mucosal erosions were found on the Bauhin's valve (Figure 1c) and ascending colon (Figure 1d). No anorectal lesions were observed. Four days after the colonoscopy, VCE was performed and found several irregular ulcers at the ileum end but no lesions were observed on its orals side. (Figure 2a–d). VCE could not clearly detect the cecal lesions because of stool and residues in the cecum(not shown). Before VCE was performed, a patency capsule was introduced for the patient and no retention was confirmed. The VCE findings were also reminiscent of CD, but no cobblestone appearance was observed. A biopsy performed during colonoscopy demonstrated granulomatous inflammation with a moderate accumulation of plasma cells and mononuclear cells (Figure 3a,b). The bacterial culture of the biopsy specimen proved the growth of YE 4 days after colonoscopy, on the same day as VCE was performed. Levofloxacine at 500 mg was administered for 7 days starting the day the results of bacterial culture were obtained, and her abdominal pain was relieved soon after. The patient has not visited our hospital for at least 2 years despite being told to come again if the symptoms recurred, so we assume there has been no recurrence.

## DISCUSSION

The pathogen YE passes into the stomach via contaminated food and invades the epithelial cells of the small intestine and the underlying Peyer patches with the help of so‐called M cells (antigen‐sampling intestinal epithelial cells).^1^ The epithelium overlying the Peyer patches has a high concentration of M cells.^1^ This is because *Yersinia* enteritis predominantly causes terminal ileitis. Given the pathogenesis of CD, *Yersinia* has been reported to induce exacerbated inflammation of the intestine in genetically predisposed subjects and may thus be a cause of CD.^6^ This theory is called the “cold chain hypothesis,” as the technological development of the refrigerator actually enabled the prolonging of bacterial life and thus increased humanity's opportunities for exposure to *Yersinia* species.^6^ Although this theory contradicts that CD is increasing in the developed countries where YE is rare but they demonstrated that an annual incidence of CD in the USA, UK, Sweden, and China strongly correlated with domestic refrigeration.^6^ Yersinia is more efficiently cultured at a low temperature of around 25°C but the real clinical setting can usually avoid false negatives by sensitive method^7^ and YE was eventually dominantly cultured in this patient.

Although *Yersinia* enterocolitis induced by YE is rare in developed countries, its differential diagnosis from CD is important based on the abdominal symptoms and endoscopic findings. More than 30 years ago, Japanese endoscopists first reported eight cases of *Yersinia* enterocolitis.^4^ According to their summary, ulcers and edema in the terminal ileum and ileocecal valve, and aphthoid ulcers in the cecum were the predominant findings by typical colonoscopy,^4^ and these findings are also frequently observed in CD.^8^ Other than CD, drug‐induced enterocolitis caused by NSAIDs or aspirin, intestinal tuberculosis, other infectious enterocolitis, and backwash ileitis associated with ulcerative colitis should also be differentiated.^8^ As a point of differential diagnosis from CD, it is known that the ulcers seen in yersinia enterocolitis are not longitudinal, namely the top of the small ridges of the ulcers are not connected to each other.^5^ For the diagnosis of *Yersinia* enterocolitis, a bacterial culture is mandatory, but a usual stool culture is normally not sensitive because regarding temperature sensitivity the optimal culture condition of *Yersinia* is around 25°C.^7^ Furthermore bacterial culture using biopsy specimens obtained during colonoscopy is more sensitive than stool culture.

To our knowledge, this is the first case report describing the VCE findings of *Yersinia* colitis. Although VCE can help confirm the spread of lesions when patients are suspected of having CD, it should be carefully introduced, as the capsule can occasionally be retained by stenotic lesions induced by CD.^9^ The risk of capsule retention should therefore be assessed using abdominal CT or enteroclysis before performing VCE.^9^ The indications for VCE to diagnose and manage CD have recently improved especially regarding better visual abilities. In addition, the development of the patency capsule, the colon capsule, and the panenteric capsule have also greatly improved the diagnosis and management of CD.^9^

The present case was first suspected of having CD because the course lasted around 1 month, and the endoscopic findings were similar to those of CD. Although *Yersinia* enterocolitis seems to be rare in countries with a well‐developed hygienic infrastructure, international human migration can facilitate the introduction of infectious diseases, thus increasing the importance of differentiating CD from *Yersinia* enterocolitis.^3^ In most cases of yersinia enterocolitis, the duration of symptoms before diagnosis varies from 1 to 2 weeks, while in a small proportion, the symptoms could be present for months before diagnosis.^1^ According to the past history, the presented case was from Vietnam 6 months before the onset of symptoms, and the incubation time seemed to be too long but no further information regarding the infection route was unfortunately available.

The significance of VCE is expected to increase in such cases. VCE was introduced in the present patient before the results of the culture were obtained. The findings of VCE showed irregular ulcers but no cobblestone‐like appearance. This is because VCE was performed 3 days after the colonoscopy, and the picture of VCE was captured without intestinal dilatation by air feeding. The evolution of artificial intelligence will further improve the accuracy of diagnoses by VCE.^10^

In conclusion, VCE could detect typical findings of *Yersinia* enterocolitis in the terminal ileum. VCE could be one of the choices to explore the endoscopic findings of such low‐burden examinations for infectious enterocolitis involving the small intestine.

## CONFLICT OF INTEREST STATEMENT

None.

## INFORMED CONSENT

Written informed consent was obtained from patients for this case report.

## References

[1] Triantafillidis JK , Thomaidis T , Papalois A . Terminal ileitis due to *Yersinia* Infection: An underdiagnosed situation. Biomed Res Int 2020; 23: 1240626. 10.1155/2020/1240626 PMC7273408 32566652

[2] Le Guern AS , Martin L , Savin C , Carniel E . Yersiniosis in France: Overview and potential sources of infection. Int J Infect Dis 2016; 46: 1–7. 26987478 10.1016/j.ijid.2016.03.008

[3] Riahi SM , Ahmadi E , Zeinali T . Global prevalence of *Yersinia enterocolitica* in cases of gastroenteritis: A systematic review and meta‐analysis. Int J Microbiol 2021; 2021: 1499869. 34512763 10.1155/2021/1499869 PMC8433020

[4] Matsumoto T , Iida M , Matsui T *et al.* Endoscopic findings in *Yersinia* enterocolitica enterocolitis. Gastrointest Endosc 1990; 36: 583–7. 2279647 10.1016/s0016-5107(90)71169-8

[5] Lee HS , Lim YJ . Capsule endoscopy for ileitis with potential involvement of other sections of the small bowel. Gastroenterology Research and Practice 2016; 2016: 9804783. 26880904 10.1155/2016/9804783 PMC4737449

[6] Hugot JP , Dumay A , Barreau F , Meinzer U . Crohn's disease: Is the cold chain hypothesis still hot? J Crohns Colitis 2021; 15: 678–86. 32949122 10.1093/ecco-jcc/jjaa192 PMC8023829

[7] Logue CM , Sheridan JJ , McDowell DA , Blair IS , Hegarty T . The effect of temperature and selective agents on the growth of *Yersinia* enterocolitica serotype O:3 in pure culture. J Appl Microbiol 2000; 88: 1001–8. 10849176 10.1046/j.1365-2672.2000.01068.x

[8] Bojic D , Markovic S . Terminal ileitis is not always Crohn's disease. Ann Gastroenterol 2011; 24: 271–5. 24713761 PMC3959324

[9] Lahat A , Veisman I . Capsule endoscopy in Crohn's disease—From a relative contraindication to habitual monitoring tool. Diagnostics 2021; 22: 1737. 10.3390/diagnostics11101737 PMC8534609 34679435

[10] Ferreira JPS , de Mascarenhas Saraiva MJDQEC , Afonso JPL *et al.* Identification of ulcers and erosions by the novel Pillcam™. Crohn's capsule using a convolutional neural network: A multicentre pilot study. J Crohn's Colitis 2022; 16: 169–72. 34228113 10.1093/ecco-jcc/jjab117

